# Gas Anesthesia Impairs Peripheral Auditory Sensitivity in Barn Owls (*Tyto alba*)

**DOI:** 10.1523/ENEURO.0140-18.2018

**Published:** 2018-11-12

**Authors:** Nadine Thiele, Christine Köppl

**Affiliations:** 1 Department of Neuroscience, School of Medicine and Health Sciences, Carl von Ossietzky University Oldenburg, 26129 Oldenburg, Germany; 2 Cluster of Excellence “Hearing4all” and Research Center Neurosensory Science, Carl von Ossietzky University Oldenburg, 26129 Oldenburg, Germany

**Keywords:** auditory brainstem response, avian, bird, isoflurane, ketamine, physiology

## Abstract

Auditory nerve single-unit recordings were obtained from two groups of young barn owls (age, between posthatching days 11 and 86) in terminal experiments under two different anesthetic regimes: ketamine (6–11 mg/kg) plus xylazine (∼2 mg/kg); or isoflurane (1–1.5%) in oxygen, delivered via artificial respiration. In a second series of minimally invasive experiments, auditory brainstem responses (ABRs) were recorded in the same four adult barn owls (*Tyto alba*; age, between 5 and 32 months) under three different anesthetic protocols: ketamine (10 mg/kg) plus xylazine (3 mg/kg), isoflurane (1–1.5%), and sevoflurane (2–3%) in carbogen. Finally, the ABR measurements on adult owls were repeated in terminal experiments including more invasive procedures such as artificial respiration and higher isoflurane dosage. The main finding was a significant deterioration of auditory sensitivity in barn owls under gas anesthesia, at the level of the auditory nerve (i.e., a very peripheral level of the auditory system). The effect was drastic in the young animals that experienced threshold elevations in auditory nerve single-unit responses of ≥20 dB. ABR thresholds assessed repeatedly in experiments on adult owls were also significantly higher under isoflurane and sevoflurane, on average by 7 and 15 dB, compared with ketamine/xylazine. This difference already occurred with minimal dosages and was reversibly enlarged with increased isoflurane concentration. Finally, there was evidence for confounding detrimental effects associated with artificial respiration over many hours, which suggested oxygen toxicity.

## Visual Abstract

**Figure:**
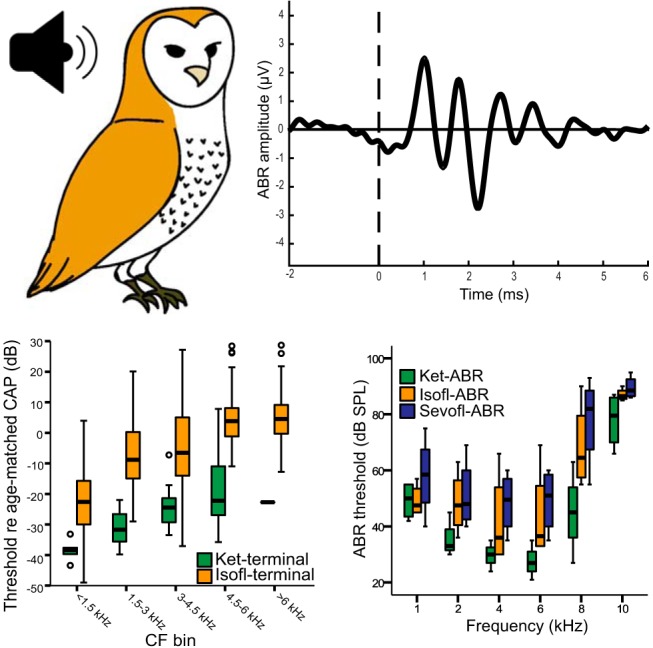

## Significance Statement

Anesthesia and analgesia are necessary for most invasive experiments. Their effects are also a concern for studying normal neural and sensory functions. We show a significant deterioration of hearing sensitivity of the auditory nerve under gas anesthesia (isoflurane or sevoflurane), compared with injection anesthesia with ketamine/xylazine, in barn owls. This generalizes similar findings across birds and mammals, and suggests that while inhalants are widely recommended as safe and easy-to-use anesthetics in veterinary contexts, they should only be used with great caution in auditory neurophysiology, even at the most peripheral level. Future important questions are whether the deterioration of sensitivity at the periphery generalizes to other senses and what the precise mechanisms are that determine the species-specific extent of sensitivity loss.

## Introduction

Anesthesia and analgesia are necessary components of most invasive physiological experiments. Yet, their very site of action, the nervous system, is a constant concern for neuroscientists who strive to study normal neural and sensory functions. Different anesthetic agents act on different neural targets and for some common anesthetic agents these are known in considerable detail at the molecular level (Lukasik and Gillies, 2003; Sonner et al., 2003; Rudolph and Antkowiak, 2004). However, due to the highly interactive nature of the intact nervous system, it remains difficult to predict their effect on a given neuronal population under *in vivo* conditions (Antkowiak, 2001; Vahle-Hinz and Detsch, 2002; Windels, 2006).

In the present study, the anesthetic regime was changed during an ongoing experimental series on the development of auditory nerve responses in the barn owl (*Tyto alba*). Previous auditory research in several laboratories had successfully used a combined ketamine/xylazine or ketamine/diazepam injection anesthesia in both adult and young owls (Cohen and Knudsen, 1995; Köppl, 1997; Keller and Takahashi, 2005; Bremen et al., 2007; Köppl and Nickel, 2007). Nevertheless, a change to isoflurane inhalant anesthesia was recommended by the consulting veterinarians, citing animal welfare concerns (Varner et al., 2004). Isoflurane and related inhalants (halogenated ethers, e.g., sevoflurane and desflurane; Campagna et al., 2003), are commonly recommended as the first choice for veterinary procedures on a wide variety of species, including birds (Korbel, 1998; Gunkel and Lafortune, 2005; Lierz and Korbel, 2012; Raftery, 2013). Frequently cited advantages are the rapid and easy control of anesthetic depth, stable anesthetic state for lengthy procedures, and rapid recovery (Keegan, 2005).

Although no specific reports were available about the effects of isoflurane on neural responses in birds, there was no reason to expect a deterioration in auditory nerve responses with gas anesthetic agents relative to ketamine/xylazine. In starlings and chickens, halothane, another inhalant anesthetic, and ketamine/xylazine were tested and compared for their effects on cochlear responses. Both types of anesthesia were found to act in an equally depressive fashion on otoacoustic emissions (produced by the hair cells of the inner ear; Kettembeil et al., 1995). Similarly, the effects of ketamine or isoflurane on auditory nerve responses in the Tokay gecko were moderately depressive and differed little from each other (Dodd and Capranica, 1992). In a bat, isoflurane had no adverse effects on otoacoustic emissions (Drexl et al., 2004), suggesting that it could even be preferable to ketamine, depending on the species. Unfortunately, it became clear during the course of this study that the change to isoflurane correlated with degraded hearing sensitivity in the barn owl. Studies in several mammalian species subsequently reported similar effects on cochlear responses (Stronks et al., 2010; Cederholm et al., 2012; Ruebhausen et al., 2012). In order to directly compare different anesthetic agents and protocols, including prolonged anesthesia and artificial respiration, which are common in invasive neurophysiology, a dedicated series of experiments on adult barn owls was finally carried out. In addition to ketamine/xylazine and isoflurane, the more recently introduced inhalant sevoflurane was then also included for testing. Sevoflurane shows a pharmacology similar to isoflurane, but induction and recovery from anesthesia are even more rapid and, as an additional benefit, it is less of an irritant to the respiratory tract (O'Keeffe and Healy, 1999; Preckel and Bolten, 2005; Flaherty, 2009; Burns, 2014).

## Materials and Methods

Experiments were carried out over a time span of several years, using two different laboratories and different recording techniques. The two experimental series will be referred to as young owls and adult owls. In young owls, compound action potential (CAP) and auditory nerve single-unit recordings were carried out. In adult owls, auditory brainstem responses (ABRs) were recorded. Within each group, different anesthetic protocols will be abbreviated as follows: ketamine-terminal and isoflurane-terminal (young owls); and ketamine-ABR, isoflurane-ABR, sevoflurane-ABR, and ABR-terminal (adult owls).

### Experimental animals

All animal procedures were performed in accordance with the German Animal Welfare Act and were approved by local authorities (permits AZ 209.1/211-2531-113/03 and AZ 33.12 42502-13/1154). In the first experimental series, 21 young barn owls of undetermined (either) sex, 18 *T. alba* and 3 *Tyto furcata* (formerly classified as *T. alba pratincola*) were used. Their hatching dates were not always known to the specific day, therefore the developmental stage was determined according to the study by Köppl et al. (2005) and was expressed as the number of days posthatching; the stage ranged from postnatal day 11 (P11) to P86. Barn owls are altricial and fledge from the nest fully grown at about P65 (Köppl et al., 2005). The ABR was recorded in four adult barn owls (*T. alba*), two females and two males, between 5 and 32 months of age and weighing between 310 and 390 g.

### Anesthesia and homeostasis

All animals were deprived of food for ∼12 h before the initiation of anesthesia. Young owls received initial doses of 10 mg/kg ketamine hydrochloride (Ketavet, Pharmacia) and 3 mg/kg xylazine hydrochloride (Rompun, BayerVital), injected intramuscularly. Young owls in the ketamine-terminal group were maintained by supplementing ketamine and xylazine as needed, usually every 30–40 min during the surgical stage and every 40–60 min during electrophysiological recordings, at dosages of 6–11 and 1.7–2.5 mg/kg, respectively. Young owls in the isoflurane-terminal group were maintained on ketamine and xylazine only for preliminary surgery, during which the trachea was cut in the neck region and intubated, and the abdominal air sac was exposed and opened. A one-way respiration system was then connected (Burger and Lorenz, 1960; Schwartzkopff and Brémond, 1963), delivering gases at a constant pressure to the tracheal tube and providing an outlet through a short tube inserted into the air sac. Spontaneous breathing immediately ceased under artificial respiration in all cases. In most experiments, pure oxygen was delivered with 1–1.5% isoflurane (Rhodia Organique Fine or Essex Tierarznei) added by a vaporizer (Vapor 19.3, Dräger), at a volume of 150–400 ml/min, depending on the size of the animal. In three experiments, carbogen (95% oxygen and 5% carbon dioxide) was used instead of pure oxygen. Respiratory gases were humidified via a wash bottle with distilled water before being delivered to the animal. All young owls in the isoflurane-terminal group also received analgesic injections of 20–50 mg/kg metamizole-sodium (Vetalgin, Intervet) at irregular intervals of 2–8 h.

Each adult barn owl was tested under three different anesthetic protocols, applied on separate days in a randomized sequence, with 1 week of recovery in between. Breathing was unaided in all cases. For the ketamine-ABR condition, owls received an initial dose of 10 mg/kg ketamine hydrochloride (bela-pharm) and 3 mg/kg xylazine hydrochloride (Medistar Arzneimittelvertrieb), i.m. Maintenance doses of 1.6–5 mg/kg ketamine and 0.6–1.8 mg/kg xylazine were given as needed, typically every 30 min. For the isoflurane-ABR condition, anesthesia was both initiated and maintained on isoflurane only. A concentration of 0.5–1.5% isoflurane (CP-Pharma Handelsgesellschaft) was added to carbogen (used to minimize the danger of apnea) by a vaporizer (Fortec, Cyprane Kneighley) and delivered via a custom-built respiration mask, at a volume of 1 L/min. For the sevoflurane-ABR condition, 2–3% sevoflurane (Ecuphar; vaporizer, Harvard Apparatus) in carbogen was delivered. At the conclusion of each experiment, the owl received a single dose of ∼0.03 mg/kg meloxicam (Boehringer Ingelheim Vetmedica), a nonsteroidal anti-inflammatory drug, for the recovery phase. In a fourth and terminal experiment, ABR measurements were repeated under different, sequentially applied protocols that included conditions closer to those of the terminal experiments on young owls. The sequence always began with ketamine/xylazine injection anesthesia, applied as before. The trachea was cut in the neck region and intubated, in preparation for later artificial respiration. However, breathing was still unaided for the first series of measurements. After that, the abdominal air sac was opened, and a one-way artificial respiration system with pure oxygen (400 ml/min) was instigated, as in young owls. After the completion of another series of ABR measurements, ketamine/xylazine anesthesia was discontinued and the anesthesia was switched to isoflurane, added to the oxygen respiration at different concentrations (1%, 2%, and back to 1%) to investigate the effect of dosage on the ABR.

All animals were killed by an overdose of sodium pentobarbital (∼100 mg/kg) at the conclusion of the terminal experiment.

The depth of anesthesia was constantly monitored via a combined EKG and muscle potential recording between needle electrodes inserted into muscles of one wing and the contralateral leg. Body temperature was held constant at 39°C by a feedback-controlled heating blanket (Harvard Systems) wrapped around the body of the animal, with the probe inserted into the cloaca. The head temperature of young barn owls was monitored separately by a small thermoprobe placed in the throat. Barn owls become homeothermic at ∼3 weeks posthatching (Shawyer, 1998), and individuals older than ∼P25 maintained a constant head temperature at 37–38°C, under these conditions. In younger animals, the unassisted head cooled significantly relative to the body, and a heat lamp was added to maintain head temperature at 35–38°C during recordings.

### Surgery

The heads of young barn owls were held firmly via an individual head band modeled from plaster-of-Paris. For CAP recordings, the round window of the inner ear on one side was exposed. For single-unit recordings from the auditory nerve, the brainstem was exposed by aspirating part of the cerebellum. Note that in these experiments, the surgical openings also vented the middle-ear space. This avoids the buildup of negative middle-ear pressure under anesthesia, which significantly reduces auditory sensitivity (for review, see Larsen et al., 2016).

For ABR measurements of adult owls, the beak of the owl was fixed in a custom-built holder. To prevent the buildup of negative middle-ear pressure under anesthesia (Larsen et al., 2016), a sterile 27 gauge cannula was inserted through the skull into the middle-ear cavity for ventilation during ABR measurements.

### Sound stimulation and electrophysiological recordings

All CAP and single-unit recording measurements took place in a custom-built double-walled sound-attenuating chamber, and ABR measurements took place in a double-walled chamber (model 1203A, Industrial Acoustics). Individually calibrated acoustic stimuli were presented through a custom-built miniature earphone and microphone system sealed into the ear canal ipsilateral to the recording electrode (ER-2 earphone, Etymotic Research; FG-23329 microphone for ABR recordings, Knowles).

For CAP recordings in young owls, a silver wire electrode, insulated except for a small bead melted at its tip, was placed onto the round window membrane, and a grounded reference electrode (Ag/AgCl pellet) was placed under the skin near the incisions made in the head. Electrode signals were amplified by a Tucker-Davis Technologies (TDT) DB4 amplifier, used at 10,000× to 100,000× amplification, 0.1 or 0.2 kHz high-pass filtering, and 15 kHz low-pass filtering (12 dB/octave Butterworth filters). Signals were then fed to a TDT AD1 analog-to-digital converter that was connected via an O1 optical interface to an AP2 signal processor interface in a personal computer. The same interface was used to synthesize the acoustic stimuli, which were then antialiased (FT6-2, TDT), variably attenuated (PA4, TDT), and fed to the earphone. Stimuli were tone pips of 20 ms duration, including 1 ms cosine-shaped rise and fall times (2 ms at 500 Hz) and were delivered at rates of 5/s. Stimuli had a fixed starting phase and equal numbers of stimulus presentations with opposite starting phase were averaged (in total, 32 or 64). Stimulus generation and CAP recording were conducted under the control of TDT software (SigGen and BioSig).

For auditory nerve single unit recordings in young owls, glass microelectrodes filled with 3 m KCL and with impedances typically between 30 and 50 MΩ were placed over the brainstem under visual control and then remotely advanced by a precision microdrive (inchworm 6000ULN, Burleigh). A grounded reference electrode (Ag/AgCl pellet) was placed under the skin near the incisions made on the head. Signals were amplified and filtered (767 electrometer, World Precision Instruments, TDT PC1 module), action potentials were threshold discriminated (SD1, TDT), and the resulting TTL pulses were fed to a TDT AP2 interface board, via an event timer (model ET1, TDT) and an analog-to-digital converter (model DD1 TDT). As above, the same interface was used to synthesize the acoustic stimuli, which were then antialiased (FT6-2, TDT), variably attenuated (PA4, TDT), and fed to the earphone. Stimulus generation and recording of the TTL pulses was under the control of custom-written software.

ABR in adult owls was recorded between two subcutaneous platinum electrodes (Grass Technologies), one placed on the vertex and one next to the left ear canal. Signals were amplified 1000× by an ISO 80 amplifier (World Precision Instruments), band-pass filtered between 0.1 and 10 kHz, and digitized by a Hammerfall DSP Multiface II Interface Card (RME Audio). Stimuli were tone bursts with 10 ms duration and 1 ms rise/fall time, and delivered at a rate of 7 bursts/s, generated by the same interface and fed to the earphone via a TDT HB7 Headphone Buffer. ABR responses were averaged over 300 stimulus repetitions. Stimulus generation and ABR recording were conducted under the control of software custom-written in MATLAB (MathWorks).

### Data analysis

CAP responses in young owls were recorded to frequencies of 500 Hz and 1 to 10 kHz in 1 kHz steps. At each frequency, responses to a range of randomly presented levels were recorded, generally in 5 dB increments, and decreased to 3 dB near threshold. CAP amplitude was defined as the difference between the first negative peak N1 and the following most prominent positive peak. Thresholds were derived from linear regression fits through the initial segment of the curve (four to six data points collected at the lowest stimulus levels), as the level eliciting a 5 µV response.

For auditory nerve single units recorded in young owls, the frequency–threshold curves were derived from responses to a matrix of tone bursts of 50 ms duration, presented randomly at different frequencies and levels, three times each, at a rate of five stimuli per second; the threshold criterion was, on average, 20 spikes/s above spontaneous rate. The spontaneous rate was estimated from the same datasets, either by counting spikes in the 50 ms window immediately before each stimulus (ketamine-terminal group) or from randomly inserted silent trials (isoflurane-terminal group). A new measure of relative sensitivity was defined that normalizes for the known threshold changes that occur with age that have been quantified for ketamine/xylazine anesthetized owls (Köppl and Nickel, 2007). Age-typical CAP thresholds can be derived for any desired age at 11 standard frequencies between 0.5 and 10 kHz, from the published fits of the CAP threshold as a function of posthatching age (Köppl and Nickel, 2007, their Fig. 8, plus data for five frequencies not shown). By linear interpolation between frequencies, age-typical CAP thresholds were then calculated for any desired frequency. The difference between the threshold of a single unit at characteristic frequency (CF) and the corresponding CAP threshold at that age and frequency was taken as a measure of relative sensitivity.

**Fig. 1.**
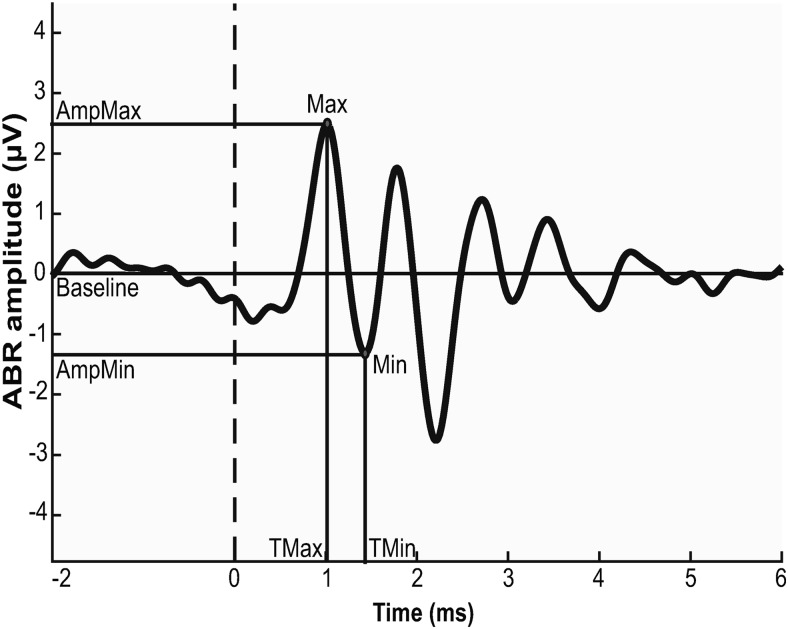
Example of a typical ABR recording from an adult owl. Shown is an average response to 300 stimuli at 2 kHz, 51 dB SPL. Only wave I was analyzed. Amplitude was defined as the difference between the first positive (AmpMax) and following negative (AmpMin) peak. Latency was defined as the latency of the first positive peak (Tmax).

In ABR recordings from adult owls, a standard set of six frequencies was tested, at 1, 2, 4, 6, 8, and 10 kHz. At each frequency, responses to a range of randomly presented levels were recorded, generally in 5 dB increments, and decreased to 3 dB near threshold (with few exceptions). ABR thresholds were identified visually, and peak-to-peak amplitude and peak latency were read out with the use of a custom MATLAB script (Fig. 1, example). To eliminate the audiogram threshold variation for graphical summaries comparing the different anesthetic protocols, ABR thresholds were normalized to the respective individual value in the ketamine-ABR condition or the ketamine condition in the ABR-terminal experiment.

### Statistical analysis

Statistical analyses were carried out with the use of PASW Statistics version 18.0.2 or SPSS Statistics versions 24 and 25 (IBM). Nonparametric measures and tests were used throughout (Table 1). A *p* value of ≤0.01 was the criterion for a significant difference. In case of multiple *post hoc* comparisons, a Bonferroni adjustment of the criterion *p* value was applied by dividing 0.01 by the number of pairwise tests required. For example, if three pairwise *post hoc* tests were performed, the Bonferroni-corrected criterion *p* value was 0.003333.

**Table 1:** List of statistical tests

Reference number	Data structure	Parameter tested	Type of test	*p* value	Figure
1	Two independent samples: ketamine-terminal (*n* = 57) isoflurane-terminal (*n* = 351)	Single-unit threshold (relative to age-matched CAP audiograms)	Mann–Whitney	**<0.001**	2*A*
2	Two independent samples: ketamine-terminal (*n* = 5) isoflurane-terminal (*n* = 49)	Single-unit threshold (relative to age-matched CAP audiograms) CF <1.5 kHz	Mann–Whitney	**0.004**	2*B*
3	Two independent samples: ketamine-terminal (*n* = 14) isoflurane-terminal (*n* = 76)	Single-unit threshold (relative to age-matched CAP audiograms) CF 1.5–3 kHz	Mann–Whitney	**<0.001**	2*B*
4	Two independent samples: ketamine-terminal (*n* = 20) isoflurane-terminal (*n* = 89)	Single-unit threshold (relative to age-matched CAP audiograms) CF 3–4.5 kHz	Mann–Whitney	**<0.001**	2*B*
5	Two independent samples: ketamine-terminal (*n* = 17) isoflurane-terminal (*n* = 86)	Single-unit threshold (relative to age-matched CAP audiograms) CF 4.5–6 kHz	Mann–Whitney	**<0.001**	2*B*
6	Five independent samples (age groups): P11 to P14 (*n* = 99) P17 (*n* = 35) P21 to 32 (*n* = 87) P35 to P40 (*n* = 17) P51 to P86 (*n* = 38)	Single-unit spontaneous discharge rate	Kruskal–Wallis	**<0.001**	
7	Two independent samples: P11 to P14 (*n* = 99) P17 (*n* = 35)	Single-unit spontaneous discharge rate	Mann–Whitney Bonferroni corrected	**<0.001**	
8	Two independent samples: P11to P14 (*n* = 99) P21 to P32 (*n* = 87)	Single-unit spontaneous discharge rate	Mann–Whitney Bonferroni corrected	**0.001**	
9	Two independent samples: P11 to P14 (*n* = 99) P35 to P40 (*n* = 17)	Single-unit spontaneous discharge rate	Mann–Whitney Bonferroni corrected	0.003	
10	Two independent samples: P11 to P14 (*n* = 99) P51 to P86 (*n* = 38)	Single-unit spontaneous discharge rate	Mann–Whitney Bonferroni corrected	0.002	
11	Two independent samples: P17 (*n* = 35) P21 to P32 (*n* = 87)	Single-unit spontaneous discharge rate	Mann–Whitney Bonferroni corrected	0.346	
12	Two independent samples: P17 (*n* = 35) P35 to P40 (*n* = 17)	Single-unit spontaneous discharge rate	Mann–Whitney Bonferroni corrected	0.992	
13	Two independent samples: P17 (*n* = 35) P51 to P86 (*n* = 38)	Single-unit spontaneous discharge rate	Mann–Whitney Bonferroni corrected	0.614	
14	Two independent samples: P21 to 32 (*n* = 87) P35 to P40 (*n* = 17)	Single-unit spontaneous discharge rate	Mann–Whitney Bonferroni corrected	0.467	
15	Two independent samples: P21 to P32 (*n* = 87) P51 to P86 (*n* = 38)	Single-unit spontaneous discharge rate	Mann–Whitney Bonferroni corrected	0.718	
16	Two independent samples: P35 to P40 (*n* = 17) P51 to P86 (*n* = 38)	Single-unit spontaneous discharge rate	Mann–Whitney Bonferroni corrected	0.826	
17	Two independent samples: ketamine-terminal (*n* = 56) isoflurane-terminal (*n* = 260)	Single-unit spontaneous discharge rate, all ages ≥P17	Mann–Whitney	**0.005**	4*A*
18	Two independent samples: ketamine-terminal (*n* = 5) isoflurane-terminal (*n* = 29)	Single-unit spontaneous discharge rate, all ages ≥P17 and CF <1.5 kHz	Mann–Whitney	0.575	4*B*
19	Two independent samples: ketamine-terminal (*n* = 14) isoflurane-terminal (*n* = 30)	Single-unit spontaneous discharge rate, all ages ≥P17 and CF 1.5–3 kHz	Mann–Whitney	0.035	4*B*
20	Two independent samples: ketamine-terminal (*n* = 19) isoflurane-terminal (*n* = 62)	Single-unit spontaneous discharge rate, all ages ≥P17 and CF 3–4.5 kHz	Mann–Whitney	0.858	4*B*
21	Two independent samples: ketamine-terminal (*n* = 17) isoflurane-terminal (*n* = 88)	Single-unit spontaneous discharge rate, all ages ≥P17 and CF 4.5–6 kHz	Mann–Whitney	0.264	4*B*
22	Two independent samples: ketamine-terminal (*n* = 53) isoflurane-terminal (*n* = 327)	Single-unit Q10 dB	Mann–Whitney	0.098	

23	Three dependent samples (*n* = 24): ketamine-ABR isoflurane-ABR sevoflurane-ABR	ABR threshold	Friedman	**<0.001**	5
24	Two dependent samples (*n* = 24): ketamine-ABR isoflurane-ABR	ABR threshold	Wilcoxon Bonferroni corrected	**<0.001**	5
25	Two dependent samples (*n* = 24): ketamine-ABR sevoflurane-ABR	ABR threshold	Wilcoxon Bonferroni corrected	**<0.001**	5
26	Two dependent samples (*n* = 24): isoflurane-ABR sevoflurane-ABR	ABR threshold	Wilcoxon Bonferroni corrected	0.076	5
27	Six independent samples: 1/2/4/6/8/10 kHz (*n* = 4 each)	ABR threshold difference: isoflurane-ABR−ketamine-ABR condition	Kruskal–Wallis	0.406	5*B*
28	Six independent samples: 1/2/4/6/8/10 kHz (*n* = 4 each)	ABR threshold difference: sevoflurane-ABR−ketamine-ABR condition	Kruskal–Wallis	0.472	5*B*
29	Three dependent samples (*n* = 16): ketamine-ABR isoflurane-ABR sevoflurane-ABR	ABR amplitudes 10 dB above threshold	Friedman	0.068	
30	Three dependent samples (*n* = 16): ketamine-ABR isoflurane-ABR sevoflurane-ABR	ABR latencies 10 dB above threshold	Friedman	0.646	
31	Two dependent samples (*n* = 24): ABR-terminal, ketamine ABR-terminal, ketamine + oxygen	ABR threshold	Wilcoxon	0.163	
32	Three dependent samples (*n* = 11): ABR-terminal, 1% isoflurane ABR-terminal, 2% isoflurane ABR-terminal, 1% isoflurane repeat	ABR threshold	Friedman	**<0.001**	6*A*
33	Two dependent samples (*n* = 11): ABR-terminal, 1% isoflurane ABR-terminal, 2% isoflurane	ABR threshold	Wilcoxon Bonferroni corrected	**0.003**	6*A*
34	Two dependent samples (*n* = 11): ABR-terminal, 2% isoflurane ABR-terminal, 1% isoflurane repeat	ABR threshold	Wilcoxon Bonferroni corrected	**0.003**	6*A*
35	Two dependent samples (*n* = 11): ABR-terminal, 1% isoflurane ABR-terminal, 1% isoflurane repeat	ABR threshold	Wilcoxon Bonferroni corrected	0.262	6*A*
36	Two dependent samples (*n* = 24): ABR-terminal, ketamine + oxygen ABR-terminal, 1% isoflurane	ABR threshold	Wilcoxon	**0.004**	
37	Two dependent samples (*n* = 24): isoflurane-ABR (normalized compared with ketamine) ABR-terminal, 1% isoflurane (normalized compared with ketamine)	ABR threshold	Wilcoxon	**<0.001**	

Column 1 shows the serial number used to refer to specific tests throughout the article. Column 2 defines the samples, and column 3 the tested parameter. Column 4 lists the specific nonparametric test used, and column 5 shows the resulting *p* value, which is highlighted in bold type if the null hypothesis was rejected. Note that the criterion *p* value was 0.01, or lower if a Bonferroni correction was applied, as indicated in Column 4. Finally, column 6 refers to the relevant figure, if applicable.

## Results

### Single-unit auditory nerve thresholds were less sensitive under isoflurane than under ketamine/xylazine

**Fig. 2.**
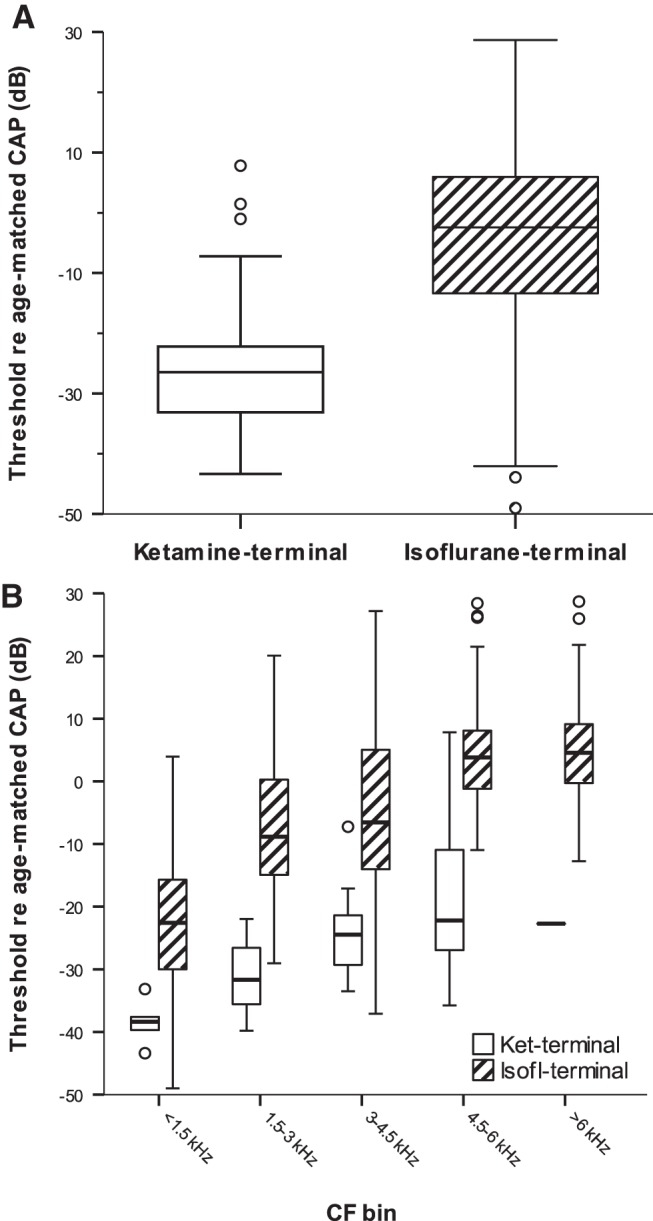
Auditory nerve single-unit thresholds were severely elevated under isoflurane. ***A***, Box plot showing thresholds (normalized to the age-matched CAP) for auditory nerve fibers recorded under ketamine/xylazine and isoflurane, respectively. ***B***, The same data, separated into 1.5-kHz-wide CF bands, for the two anesthetic conditions. Empty boxes represent data for the ketamine-terminal conditions, and hatched boxes represent data for the isoflurane-terminal condition. Note that thresholds under isoflurane were significantly higher. Boxes and whiskers indicate the interquartile ranges and 1.5 times the interquartile ranges, respectively. Horizontal lines within boxes indicate medians, and circular symbols indicate outliers that lie beyond 1.5 times the interquartile range.

A total of 57 auditory nerve fibers were recorded in the ketamine-terminal condition, from owls aged P17 to P36, and 360 auditory nerve fibers in the isoflurane-terminal condition, from owls aged P11 to P86. As absolute sensitivity is known to change within these age brackets (Köppl and Nickel, 2007), the difference between the threshold of a single unit and the CAP threshold typical for that age and frequency was defined as a measure of relative sensitivity (see Materials and Methods). This eliminated the known maturational changes of auditory thresholds, allowing for the identification of other factors influencing hearing sensitivity. There was a significant difference in relative sensitivity between auditory nerve fibers recorded under the two anesthetic protocols (Table 1, References 1). Median values were −2.4 dB for the isoflurane-terminal condition and −26.5 dB for the ketamine-terminal condition (Fig. 2*A*). This difference held across frequencies when tested separately for different characteristic frequencies, binned into 1.5-kHz-wide bands. Median relative thresholds for the isoflurane-terminal condition were between 17 and 26 dB higher than those recorded under the ketamine-terminal condition (Table 1, References 2–5, Fig. 2*B*; note that for CFs >6 kHz, the sample for the ketamine-terminal group was insufficient for a meaningful test).

### CAP thresholds were less sensitive under isoflurane than under ketamine/xylazine

**Fig. 3.**
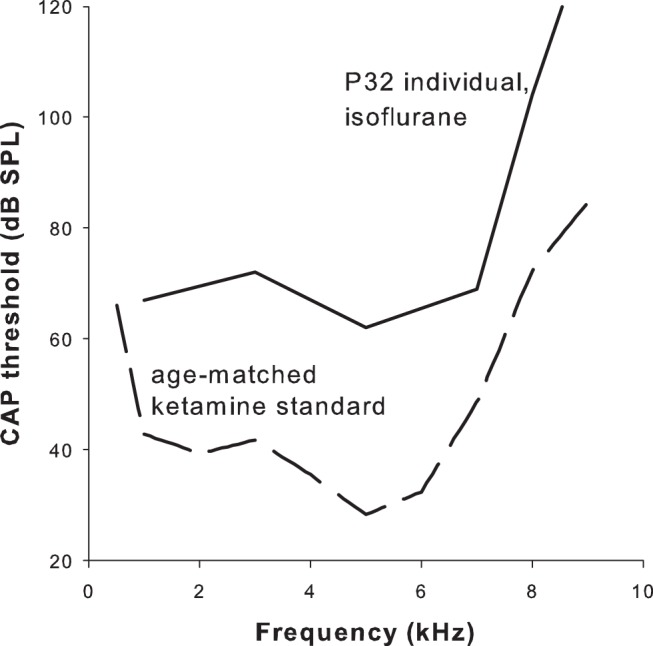
CAP thresholds were severely elevated under isoflurane. CAP threshold audiogram of an individual aged P32, under isoflurane anesthesia (solid line). For comparison, the average CAP audiogram for P32 owls under ketamine/xylazine anesthesia is also shown (dashed line; after Köppl and Nickel, 2007).

CAP thresholds were obtained in only one young owl in this study. Nevertheless, this case is included here as it validated the principal assumption that CAP and single-unit thresholds are tightly correlated. Single-unit data and CAP recordings were obtained in the same individual, aged P32, under isoflurane anesthesia. The median single-unit sensitivity relative to the animal’s own CAP audiogram was −23 dB (*n* = 7; CFs, 2.6–4.4 kHz), while it was +10.2 dB relative to the age-matched CAP threshold under ketamine/xylazine anesthesia (Köppl and Nickel, 2007). This individual thus represented a drastic case of threshold loss (Fig. 3). Furthermore, the direct reference of single-unit thresholds to the animal’s own CAP thresholds supports the notion derived from the population data that single-unit thresholds fall, on average, ∼20 dB below the CAP thresholds obtained under comparable conditions. This is typical for birds in general (Köppl and Gleich, 2007).

### Spontaneous discharge rates and frequency tuning were much less affected

**Fig. 4.**
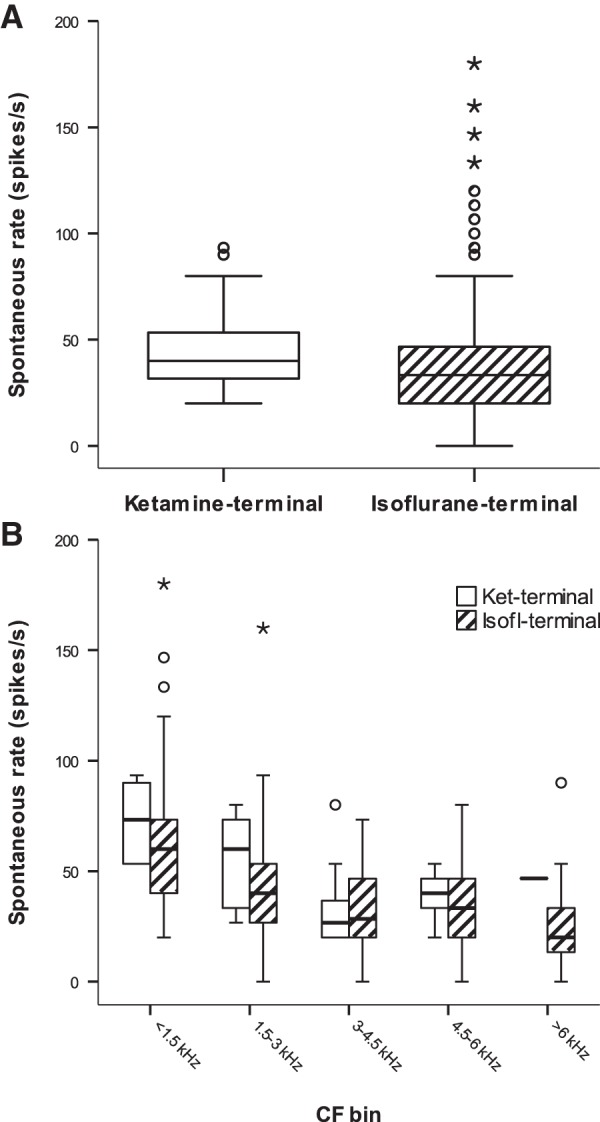
Auditory nerve spontaneous discharge rates were mildly depressed by isoflurane. ***A***, Box plot showing overall spontaneous discharge rates for auditory nerve fibers recorded under ketamine/xylazine and isoflurane, respectively. The rates under isoflurane were significantly lower. ***B***, The same data, separated into 1.5-kHz-wide CF bands, for the two anesthetic conditions. Empty boxes represent data for the ketamine-terminal conditions, and hatched boxes represent data for the isoflurane-terminal condition. Boxes and whiskers indicate the interquartile ranges and 1.5 times the interquartile ranges, respectively. Horizontal lines within boxes indicate medians, and circular symbols and stars indicate outliers that lie beyond 1.5 times (circles) or beyond 3 times (stars) the interquartile range.

As a measure of discharge activity of auditory nerve single units, spontaneous rates were evaluated for effects of the anesthetic protocol. First, data were examined for confounding age-related maturation of spontaneous rate. There was evidence for lower spontaneous rates in very young owls, aged P11 to P14, but no further changes in animals older than that (Table 1, References 6–16). To minimize maturation effects, the comparison between the anesthetic groups was therefore restricted to owls aged P17 and older. In these groups, spontaneous rates were significantly lower in the isoflurane-terminal group (Table 1, Reference 17, Fig. 4*A*). Median values were 40 spikes/s in the ketamine-terminal group and 33.3 spikes/s in the isoflurane-terminal group. Since spontaneous rates are, in addition, known to vary with CF (Köppl, 1997), the data were further examined separately, for 1.5-kHz-wide CF bands. Here, the difference between the anesthetic regimes did not hold for any CF band (Fig. 4*B*, Table 1, References 18–21; note that for CFs >6 kHz, the sample for the ketamine-terminal group was insufficient for a meaningful test). Together, isoflurane thus appeared to have a mildly depressive effect on spontaneous rates when compared with data from ketamine/xylazine-anesthetized juvenile owls.

The quality of frequency tuning, expressed as Q_10dB_, was not consistently affected by the anesthetic protocol. An overall comparison of Q_10dB_ values between the ketamine-terminal and the isoflurane-terminal groups revealed no significant difference (Table 1, Reference 22).

### ABR thresholds were less sensitive with gas anesthesia compared with ketamine/xylazine

**Fig. 5.**
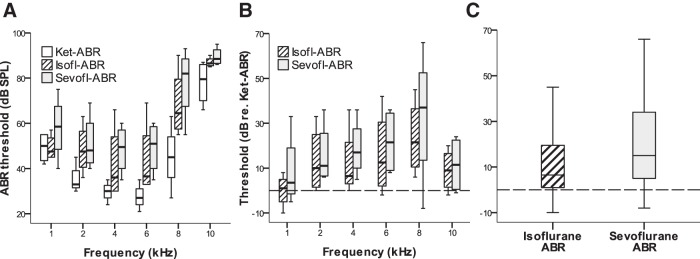
ABR thresholds were elevated under gas anesthesia. ***A***, Box plot showing ABR thresholds as a function of frequency, for the same four adult individuals, tested with different anesthetic protocols in successive experiments. ***B***, The same data, with thresholds now normalized to the values at the respective frequency for the ketamine-ABR condition; as a visual reference, the dashed line indicates the reference condition. Note that the minor variation across frequencies was not significant (Table 1, References 27 and 28). Therefore, ***C*** then shows an overall comparison between anesthetic conditions. Thresholds for either the isoflurane-ABR or sevoflurane-ABR condition were significantly higher than thresholds for the ketamine-ABR condition (Table 1, References 24 and 25). Thresholds for the ketamine-ABR condition are shown as empty boxes, for the isoflurane-ABR condition as hatched boxes, and for the sevoflurane-ABR condition as gray boxes. Boxes and whiskers indicate the interquartile ranges and 1.5 times the interquartile ranges, respectively. Horizontal lines within boxes indicate medians. There were no outliers beyond 1.5 times the interquartile ranges.

Four adult owls were tested under three anesthetic protocols each: ketamine-ABR, isoflurane-ABR, and sevoflurane-ABR. Importantly, the sequence of testing was randomized and different for each owl. ABR audiograms showed a similar overall shape for all conditions, suggesting that the basic relationship between ABR threshold and frequency was not affected (Fig. 5*A*). However, thresholds differed significantly between the conditions (Table 1, Reference 23). Specifically, thresholds in the ketamine-ABR condition were significantly lower than thresholds for either the isoflurane- or sevoflurane-ABR condition (Table 1, References 24 and 25). There was no significant difference between ABR thresholds obtained with the two anesthetic gases (Table 1, Reference 26). Threshold differences to the respective threshold in the ketamine-ABR condition showed no significant frequency dependence (Table 1, References 27 and 28, Fig. 5*B*). Overall, thresholds under isoflurane showed a median elevation of 7 dB, thresholds under sevoflurane showed a median elevation of 15 dB compared with the ketamine-ABR condition (Fig. 5*C*).

The amplitude and latency of ABR wave I were unaffected by the anesthesia protocol. Of course, amplitude increased and latency decreased with increasing sound level. Therefore, this comparison was carried out at a relative level of 10 dB above the respective ABR threshold (Table 1, References 29 and 30).

**Fig. 6.**
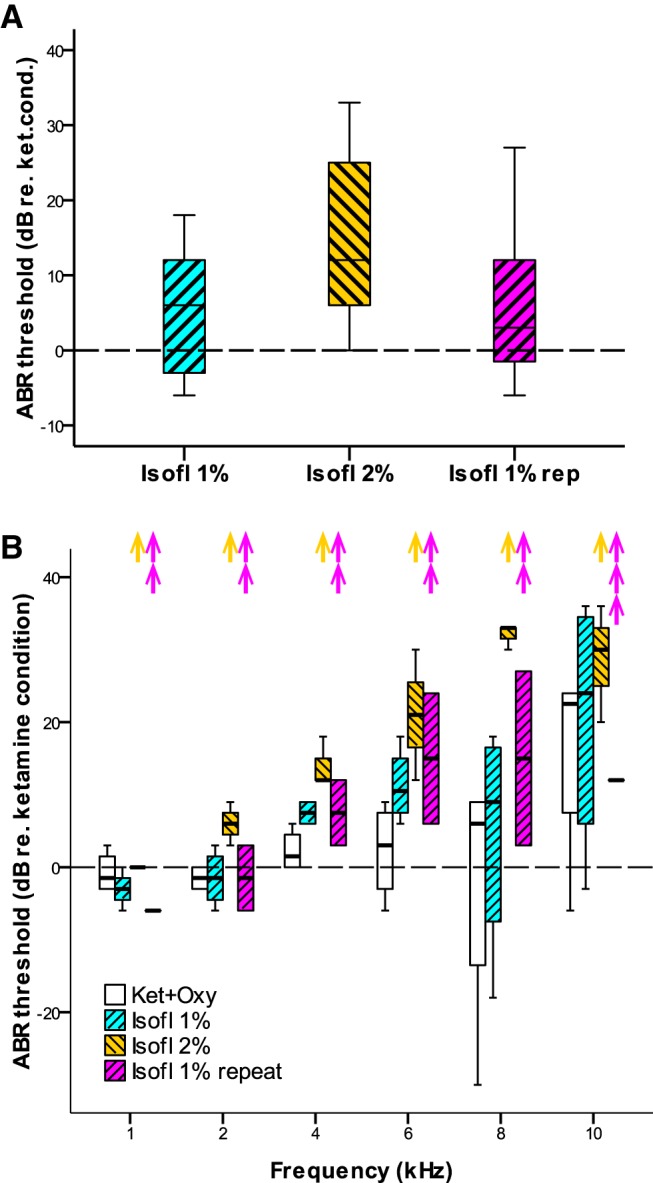
***A***, Isoflurane dose dependence of ABR thresholds. Box plot showing thresholds for different isoflurane concentrations tested sequentially in ABR-terminal experiments. Thresholds under 2% isoflurane were significantly elevated relative to those under both 1% conditions (Table 1, References 32–35). ***B***, Gradual threshold deterioration with time in terminal ABR experiments. Shown are ABR thresholds normalized to the values for the initial ketamine/xylazine condition (animal breathing air unaided), separated according to frequency, for all conditions tested sequentially. Note that the number of data points contributing to each box now varies; upward arrows indicate data that dropped out because the threshold exceeded the limit of the sound system and thus could not be determined. Note also that high frequencies appear to be affected more. Thresholds obtained under ketamine + artificial oxygen respiration are shown as empty boxes, for the 1% isoflurane condition as hatched blue boxes, for the 2% isoflurane condition as hatched yellow boxes, and for the repeated 1% isoflurane condition as hatched magenta boxes. Boxes and whiskers indicate the interquartile ranges and 1.5 times the interquartile ranges, respectively. Horizontal lines within boxes indicate medians. There were no outliers beyond 1.5 times the interquartile ranges.

In a final, terminal experiment, each adult owl was tested with more invasive protocols to assess confounding factors such as artificial respiration and variable inhalant concentration. There was no significant change in ABR thresholds after switching from unassisted breathing through a tracheotomy to artificial respiration with oxygen, both still under ketamine/xylazine anesthesia (Table 1, Reference 31). Next, the anesthetic protocol was switched to isoflurane and stepped from 1% to 2% and back to 1%, with a minimum equilibration time of 15 min before measurements were obtained after a change in gas concentration. This revealed a significant effect of isoflurane dosage (Table 1, Reference 32, Fig. 6*A*). Increasing isoflurane from 1% to 2% resulted in a significant rise of ABR thresholds (Table 1, Reference 33, Fig. 6A), which was reversible upon a return to 1% (Table 1, References 34 and 35, Fig. 6*A*). Note that, unfortunately, the full sequence of tests could be completed for only two owls. At the point of the initial switch to 1% isoflurane, the full sample from all four owls could still be obtained and showed a significant elevation of thresholds relative to the ketamine condition with artificial respiration tested immediately before (Table 1, Reference 36), thus confirming the principal effect observed in the previous ABR experiments with the same individuals. As the terminal experiments progressed, however, an unexplained, gradual, and irreversible loss of sensitivity occurred at different times for different owls that, in addition, appeared to affect the higher frequencies more (Fig. 6*B*). Some thresholds exceeded the maximum SPL of the sound system, such that the sample gradually diminished (Fig. 6*B*). The cause for this deterioration, which appeared unrelated to the anesthetic protocol is unknown and will be discussed below in the section about confounding factors. Importantly, we observed no changes to the frequency of heart beat or the shape of the EKG waveform that might correlate with the loss of sensitivity.

### Interaction with type of respiration

The above observations suggested significant factors that influence auditory sensitivity, in addition to the anesthetic agent. This prompted us to compare thresholds obtained with two types of respiration, both under isoflurane anesthesia. Indeed, thresholds in the isoflurane-ABR condition and the ABR-terminal-isoflurane 1% condition, both normalized to the respective ketamine thresholds, differed significantly (Table 1, Reference 37). The main difference was the type of respiration: unassisted breathing of 0.5–1.5% isoflurane delivered in carbogen versus artificial respiration with 1% isoflurane delivered in pure oxygen. In the condition we normalized to, owls always breathed normal room air unassisted. While both isoflurane conditions resulted in a threshold loss (as already shown above), the loss was relatively greater when breathing carbogen unaided, by a median of 1 dB.

## Discussion

The main finding of the present study was a significant deterioration of auditory sensitivity in barn owls under gas anesthesia. The effect was drastic in young animals that, compared with age-matched individuals that were anesthetized with a combination of ketamine and xylazine, experienced threshold elevations in auditory nerve single-unit responses of ≥20 dB. Consistent with this, ABR thresholds assessed repeatedly in experiments on adult owls were also significantly lower under ketamine/xylazine anesthesia, compared with gas anesthesia with both isoflurane and sevoflurane. Importantly, this difference already occurred with minimal dosages and was reversibly enlarged with increased isoflurane concentration. Finally, there was evidence for confounding detrimental effects associated with the respiration mode.

### Reports of anesthetic effects on peripheral auditory responses across species

Evidence that inhalant anesthetics adversely affect cochlear sensitivity has accumulated in recent years for mammals also. In the guinea pig, isoflurane was shown to have a dose-dependent depressive effect on several auditory evoked potentials [CAP, cochlear microphonic (CM) potential, and ABR; Stronks et al., 2010). Thresholds, amplitudes, and neural latencies were all negatively affected. The effects were most pronounced at higher frequencies ≥8 kHz, where CAP thresholds were elevated by ∼10-15 dB. Stronks et al. (2010) referenced the measurements obtained under isoflurane to the awake condition, which does not necessarily suggest that isoflurane acts worse than other anesthetics. In rat and mouse, ABR thresholds were directly compared between anesthesia with isoflurane and with ketamine/xylazine, and they were found to be relatively elevated under isoflurane (Cederholm et al., 2012; Ruebhausen et al., 2012). Furthermore, in the gerbil, ABR thresholds under ketamine/xylazine were not significantly different from those in the awake condition (Smith and Mills, 1989). Together, these studies strongly support a differentially detrimental action of the gas anesthetic, very similar to the present findings in the barn owl.

However, there is clearly also species-specific variation in the sensitivity to different anesthetics, and gas anesthetics do not fare universally worst. In the starling, a small songbird, CAP amplitudes were depressed under halothane anesthesia, at concentrations as low as 0.5%, compared with the awake state (Kettembeil et al., 1995). The same study, however, observed equally depressive effects of ketamine/xylazine and halothane anesthesia on otoacoustic emissions, which reflect the responses of sensory hair cells, in both starlings and chickens. Since neural responses were not obtained under ketamine/xylazine, it thus remained unclear whether they might be differentially affected. In a systematic comparison of auditory nerve single-unit responses under different anesthetic protocols in a lizard, the Tokay gecko, Dodd and Capranica (1992) found significantly elevated thresholds under both isoflurane and ketamine, compared with pentobarbital anesthesia. Thus, in the gecko, too, ketamine had similarly degrading effects compared with isoflurane, albeit comparatively highly dosed at 440 mg/kg (Dodd and Capranica, 1992). Furthermore, in the Tokay gecko, in contrast to the present study in the owl, auditory nerve discharge rates were also severely and differentially depressed under the different anesthetic conditions: the highest rates were observed under pentobarbital, followed by ketamine, with isoflurane having the lowest rates (Dodd and Capranica, 1992).

Finally, there is conflicting evidence regarding the effects of gas anesthetics on the responses of sensory hair cells, specifically the outer hair cells of the mammalian cochlea, measured as otoacoustic emissions. In humans, several studies reported a selectively depressive effect of gas anesthetics on evoked emissions (Ferber-Viart et al., 1998; Ropposch et al., 2014; Gungor et al., 2015). However, it is currently unclear whether this is a truly pharmacological effect on the cochlea or may be a secondary consequence of changes in arterial blood pressure. In a bat species, isoflurane was shown to have the opposite effect (i.e., increased emission amplitudes; Drexl et al., 2004). It was suggested that this may reflect disinhibition through inactivation of the olivocochlear efferent input (see also next section).

### Possible mechanisms of isoflurane and sevoflurane action

The mechanisms that produce general anesthesia at the systems level are still poorly understood (Rudolph and Antkowiak, 2004; Ishizawa, 2007). The cellular sites of action commonly involve ion channels and neurotransmitter receptors that are widely expressed in the CNS and should thus act at all levels. Nevertheless, as a general rule, a gradual effect is observed, such that higher-level cognitive functions are impaired at lower anesthetic concentrations than motor functions, early visual processing, or basic homeostatic physiology (Campagna et al., 2003; Rudolph and Antkowiak, 2004; Ishizawa, 2007). This suggests that while the cellular sites of action may be similar, higher centers tend to show the combined result of direct anesthetic action and cumulative effects in neural networks. This also promotes the common assumption that general anesthesia, when appropriately dosed, should not significantly affect primary sensory processes. Therefore, the pronounced effect of isoflurane and sevoflurane at the most peripheral levels of the auditory system, the hair cells and auditory nerve, is surprising.

Isoflurane and probably all inhalant anesthetics belonging to the halogenated alkanes and ethers, such as halothane and sevoflurane, have several known target sites of action, all of which are predicted to suppress neural activity (for review, see Campagna et al., 2003; Rudolph and Antkowiak, 2004; Burns, 2014). They suppress excitatory transmission through the inhibition of glutamate receptors, both the NMDA and AMPA subtypes, and through inhibition of nicotinic acetylcholine (ACh) receptors. Conversely, they enhance inhibitory transmission through the potentiation of GABA_A_ and glycine receptors. In contrast, the sites of ketamine and xylazine action are more restricted. Ketamine predominantly inhibits NMDA-type glutamate receptors and nicotinic ACh receptors, both normally excitatory (Rudolph and Antkowiak, 2004). Xylazine is a known agonist of the α_2_-adrenergic receptor (Lierz and Korbel, 2012), which is best known for mediating the inhibition of sympathetic activity of the autonomic nervous system.

Considering these anesthetic profiles, where are the potential sites of action at the cochlear level? Glutamate receptors of all ionotropic subtypes are typically found on the afferent terminals connecting to vertebrate hair cells (Eatock and Lysakowski, 2006). In the mammalian cochlea, the functionally predominant receptors are those of the AMPA subtype (Ruel et al., 2007; Glowatzki et al., 2008). Assuming the same for birds, isoflurane and sevoflurane are indeed predicted to have a potentially larger direct impact on auditory afferents than ketamine. However, in the present study, the observed effect was curiously specific to auditory thresholds and affected discharge rates only mildly. This is not obviously compatible with a general suppressive effect on the auditory afferents.

There is currently no evidence for the inhibitory neurotransmitters GABA and glycine in the avian cochlea (for review, see Köppl, 2011), so these are not likely to be potential mediators of the observed threshold shifts in birds. Remaining possible sites of action are the cholinergic terminals of efferent fibers to the cochlear hair cells (Köppl, 2011). Indeed, an inhibitory effect of isoflurane on these has been indirectly suggested for mammals (Drexl et al., 2004). Depending on the subtypes of efferents activated, such an enhancement could conceivably mediate a suppressive effect on auditory nerve afferents. However, the pharmacology of the hair cell ACh receptor is of an unusual nicotinic subtype (Katz et al., 2011), and there have been no direct tests for interactions with any anesthetic agent.

In summary, selectively depressive effects of gas anesthetics relative to ketamine on cochlear neural responses are consistent with their wider spectrum of known cellular interaction sites, specifically their inhibitory action on ionotropic glutamate receptors. However, the specific pattern of a drastic threshold shift without a comparable effect on discharge rates or frequency tuning, does not fit any straightforward predictions. Importantly, it may point to additional, confounding effects associated with prolonged and invasive protocols.

### Confounding factors

The anesthetic agents were not the only difference between the experimental groups in our initial study with young barn owls. Young owls of the isoflurane-terminal group were artificially respirated with oxygen or carbogen, while the ketamine-terminal group breathed normal air unaided. Therefore, in the follow-up study on adult owls, each individual was tested repeatedly under otherwise identical conditions. This confirmed the specific, detrimental effect of gas anesthesia. However, the threshold difference relative to ketamine anesthesia was smaller for the group of adult owls, opening several possibilities for confounding effects. These were addressed in the terminal experiments with adult owls.

Similar to previous studies (Kettembeil et al., 1995; Stronks et al., 2010), it was shown that the inhalant anesthetic elevated peripheral auditory thresholds in a dose-dependent manner. Thus, dosage is a likely confounding factor in experiments using more invasive procedures, such as single-unit recordings, which tend to require higher anesthetic dosages.

Mode of respiration is another possible confounding factor that was tested. Breathing carbogen unassisted appeared to result in additional sensitivity losses, over and above those related to isoflurane anesthesia, when compared with artificial respiration with pure oxygen. We had chosen carbogen (as opposed to pure oxygen) in the experiments where the owls breathed the isoflurane mixture unassisted, to minimize the danger of apnea. CO_2_ is known to be an important respiratory regulator, and low partial pressure of CO_2_ (p_CO2_) tends to depress respiration (Powell, 2015). Our results, however, suggest that this was misguided and instead resulted in a slight additional loss of sensitivity. An obvious explanation for this effect is lacking. We do not consider hypoxia very likely, since carbogen still contains 95% oxygen (i.e., a concentration far above normal air). Similarly, artificial respiration with pure oxygen is unlikely to cause hypoxia but still appeared to cause a decline in auditory sensitivity over time. While no consistent, immediate deterioration associated with the switch to artificial oxygen respiration was found, unexplained drastic threshold losses occurred beyond 4–5 h in adult owls receiving artificial oxygen respiration. This is reminiscent of the significant further deterioration within 1 h reported for mice under isoflurane (but not ketamine) anesthesia, breathing oxygen unaided (Cederholm et al., 2012). Such a time-dependent deterioration could also have been a confounding factor for the auditory nerve single-unit thresholds reported here, as these measurements typically only began with a substantial delay after anesthetic induction, due to prolonged surgery. Together, these observations point to additional, detrimental changes in the long term that would not be observed in short-term experiments such as minimally invasive ABR measurements or most veterinary procedures. Importantly, there were no indications from our EKG monitor that the state of the animals may have been compromised.

Interestingly, a study (Jaensch et al., 2001a,b) exposing awake budgerigars to pure oxygen over variable times, from 3 h to 3 d, found indicators of oxygen stress by reactive oxygen species from the shortest exposure and, with longer exposure, additional evidence of pulmonary inflammation due to oxygen toxicity. Although their birds showed no outward signs, the authors concluded that “in a clinical setting, elevated inhalant oxygen tensions should be provided to birds with caution, especially if prolonged or repeated exposure is anticipated.” This suggests that there is a point where the delivery of pure oxygen changes from being beneficial to damaging. This point may well be reached earlier in healthy subjects, as typically used in a neurophysiological research setting, compared with veterinary patients. Data on respiratory or other metabolic parameters, such as arterial partial pressure of O_2_, p_CO2_, or blood pressure and pH, would thus be desirable but were not monitored in any of the auditory studies. Unfortunately, they are known to be difficult to obtain for small birds with body weights <400 g (Desmarchelier et al., 2007; Lierz and Korbel, 2012).

### General implications for invasive neurophysiology

The above findings suggest that despite their advantages regarding animal welfare, isoflurane and related inhalant anesthetics are not the first choice for experiments in sensory and neurophysiology. The present article focused on auditory physiology; however, there is similar evidence, for example, for the visual cortex (Michelson and Kozai, 2018). Researchers should be aware that sensory responses may already be depressed at the most peripheral levels, as now shown clearly for the auditory nerve. Effects on peripheral responses must also be expected to propagate through brain nuclei. Indeed, reports of reduced sensitivity and neural activity under isoflurane anesthesia in mammalian auditory cortex (Cheung et al., 2001; Noda and Takahashi, 2015) are plausibly explained by the extent of peripheral effects observed here and in other studies. One should also be aware, however, that the brain is not organized along one-dimensional hierarchies. For example, the auditory systems of both birds and mammals show a multitude of ascending and descending interconnections (Smith and Spirou, 2002; Bolhuis et al., 2010). This makes it difficult to predict to what extent a given peripheral impairment will be evident in higher-order responses. Direct actions of inhalants on higher-order neurons may add to any inherited effects.

The degradation of auditory sensitivity was dose dependent in the present and previous studies. In order to minimize the detrimental effects of inhalant anesthetics, their dosage should therefore be individually adjusted as low as possible. In the veterinary literature, one recommended way to reduce the dose further is to administer a combination of isoflurane (or related inhalants) and nitrous oxide (Korbel, 1998). However, regarding sensory responses, we caution that this may trade one evil for an even worse one. Sloan et al. (2010) compared ABR, as well as somatosensory and visual evoked responses in the baboon, measured under anesthesia with different proportionate mixtures of isoflurane and nitrous oxide. They found evidence for a synergistic action of the two agents (i.e., the combination produced more drastic effects on the sensitivity and latency of the responses than predicted from a simple addition of the individual effects of isoflurane and nitrous oxide). Consistent with that, Anderson and Young (2004) found that adding nitrous oxide in order to reduce the isoflurane necessary did not avoid the depressive effects on sensitivity, discharge rate, and extent of inhibition in cat dorsal cochlear nucleus units.

Finally, there was clear evidence for additional detrimental effects on auditory sensitivity related to the modes of respiration. While it may seem trivial that adequate respiration needs to be provided, the observed effects could not simply be traced to hypoxia. Instead, we obtained tentative evidence for oxygen toxicity developing over time and, furthermore, observed an unexplained deterioration when the owls breathed carbogen. Whether these effects occur only in conjunction with isoflurane, or may at least be exacerbated by it, remains to be shown. Our results suggest that for prolonged experiments with healthy experimental animals, normal air is the best option.

## Synthesis

Reviewing Editor: Darcy Kelley, Columbia University Decisions are customarily a result of the Reviewing Editor and the peer reviewers coming together and discussing their recommendations until a consensus is reached. When revisions are invited, a fact-based synthesis statement explaining their decision and outlining what is needed to prepare a revision will be listed below. The following reviewer(s) agreed to reveal their identity: Joe Schumacher.

nesthesia and analgesia are essential for invasive physiology experiments to ensure humane animal use and well controlled recording conditions, but they alter neuronal function through several mechanisms. Thus, it is important to characterize the impact of anesthetic agents on the function of neural systems to better interpret physiological data. Physiologists often avoid the use of isofluorane due to reductions in baseline and evoked neural activity at moderate doses. That this seems particularly strong in mammalian cortex perhaps contributes to the prevailing incorrect assumption mentioned by the authors that isoflurane may be less of a hindrance for studies of neural activity closer to the sensory epithelium. This article demonstrates that in the avian auditory system this is not the case, and that gas anesthesia can dampen responsiveness at the periphery. Both isoflurane and sevoflurane introduce a larger rise in auditory threshold than ketamine/Xylazine at low doses. In some cases, the difference was more than 20 dB. In addition, larger doses of gas anaesthetic could also lead to irreversible loss of sensitivity possibly due to oxygen toxicity during artificial respiration could have occurred. The paper is well written and the data are solid and are valuable when assessing effects of anaesthesia. The use of birds is adequate and also allows conclusion on other vertebrates like mammals. The data also seem to be quite useful for discussions with a nonscientific clientele (veterinarians) when advantages or disadvantage of different anaesthesia regimes have to be addressed. While, in general, the experimental methods are appropriate for the questions addressed here. there are instances of questionable statistical approaches that the authors should address.

Major comments: 

Introduction 

l.58 It is not clear why breaks in the experimental protocol are a necessary disadvantage. The liquid anaesthetic could have been administered continuously with a pump (as for gas anaesthesia) to avoid breaks. 

Materials and Methods

l.130 and following ... The reference to ketamine- isoflurane etc. - recovery conditions is not clear. The animals were anaesthetized and were not in a phase of recovery from anaesthesia. At some point they had recovered, your data were obtained during anaesthesia. It indeed could be the case that some of anesthetics have longer lasting effects that persist into the recovery phase ]but this does not appear to be the focus of this study. To avoid ambiguity, omit the ‘recovery’ terms. 

L.159 How did the EKG rates change during anesthesia? 

l.175 Here, or elsewhere, briefly describe the effect of non-venting the middle ear on thresholds

l.199 and following ... CAP and single unit recordings use a 5 Hz stimulus repetition rate, is this also the case for ABR recordings? Did the scope of effects of anaesthesia depend on repetition rate? If auditory nerve or other peripheral adaptive phenomena differ for different anesthetics, repetition rate would influence results. It would also be valuable to assess whether evoked spike rates and latencies (response strength at some dB above threshold) differed between anesthesia groups. Results may or may not be consistent with the authors' findings on ABR amplitude and latency. 

l.239 It is not clear what advantages the ‘new’ measure of relative sensitivity - using Cap thresholds as reference for single unit thresholds - provides. Cap threshold data are obtained by much higher SPLs than the single unit thresholds Actual single unit thresholds in dB SPL would provide additional information on absolute auditory sensitivity in barn owls.

Statistical analyses: Spontaneous firing rates were significantly lower for the isoflurane-terminal group than the ketamine-terminal group overall, a finding of substantial relevance to this study. The statistical analysis reported in Fig. 4 seek to undercut this finding by breaking the data down into bins by characteristic frequency. This grouping is justified by pointing out that spontaneous rates vary by CF, but sampling also varies between CF bins. The justification for an unconventional statistical approach is not clear. In Fig. 4, it is not clear how strong the main effect for anesthetic groups is; statistical comparisons between groups in smaller CF-specific samples could be corrupting this main effect. Notably individual Mann-Whitney U tests for comparisons between groups used as N as small 1 cell in the case of the high CF tuning group. It is likely that some of these tests lack the statistical power necessary to come to a reasonable CF-specific conclusion. A larger binning of CFs to improve sample sizes might help. Otherwise,main effect for the anesthetic group and the relatively large median decrease for the iso animals should be reported. A multivariate approach with consideration of multiple comparisons is also an alternative approach. A similar criticism is applicable to the statistical analysis performed on ABR thresholds reported in figure 6. 

Discussion

Young owls in the ketamine-terminal group were induced and maintained under anesthesia with simple injections, while the isofurane-terminal animals underwent a tracheotomy and artificial respiration. The authors are correct to point out that it is not simply the drugs that differed in the two groups, but also the surgical procedures and maintenance of the plane of anesthesia that differed. Furthermore, most animals in the inhalant group were respirated with pure oxygen while the injection group breathed room air. To address the potential confounding factor of long-term exposure to pure oxygen, it would be valuable to see a comparison of the carbogen and oxygen groups.

The paper's impact would be strengthened by considering these results in the broader context of neuronal processing at multiple levels of sensory hierarchies. For instance, what is the predicted impact of these levels of sensitivity loss on downstream regions, and what new insights does this potentially give us into the interpretation of previous data across avian or even mammalian species? Finally, the authors are advised to address the bottom line here: what does the relative impact of gas anesthesia on neural responses say about its use in neurophysiology? Given that it has an observable dose-dependent effect, are there other ways of safely and humanely administering, for example, isoflurane while also mitigating depression of neural responses? One common approach is the combination of isoflurane and nitrous oxide, which allows for a deeper plane of anesthesia with less isoflurane and oxygen toxicity. Comparing this anesthetic regime with the others in this study would be highly valuable to the field.

Minor comments: 

Line 42: “given neuronal population” 

Line 262: The authors self-assign a significance criterion of p<0.01, but report a significant difference of P<0.03 in the text when describing Table 1, ref. #17. 

Line 319-20: The wording here is a little confusing. The ABR audiogram shapes were the same or the relationship between ABR threshold and Frequency had the same shape?

## Author Response

Reviewer comments

Author responses (line numbers refer to the file with revisions marked).

Reviewing Editor: Manuscript Instructions

The sex of the species studied is not mentioned in the materials and methods section. Please mention “male”, “female”, “x males and x females”, “of either sex”.

This information has been added

eNeuro is pleased to be part of the Resource Identification Initiative.....

We are aware of this initiative and have already made every effort to comply. However, we were unable to find RRIDs for the animals, reagents etc. used in our study. Therefore, our materials are listed in conventional form.

Synthesis of Reviews: Major comments: Introduction

l.58 It is not clear why breaks in the experimental protocol are a necessary disadvantage. The liquid anaesthetic could have been administered continuously with a pump (as for gas anaesthesia) to avoid breaks.

This statement was deleted.

Materials and Methods

l.130 and following ... The reference to ketamine- isoflurane etc. - recovery conditions is not clear. The animals were anaesthetized and were not in a phase of recovery from

anaesthesia. At some point they had recovered, your data were obtained during anaesthesia.

It indeed could be the case that some of anesthetics have longer lasting effects that persist into the recovery phase ]but this does not appear to be the focus of this study. To avoid ambiguity, omit the ‘recovery’ terms.

The confusing terms were replaced throughout and are now referred to as “ABR”

conditions.

L.159 How did the EKG rates change during anesthesia?

The EKG was continuously monitored on an oscilloscope display during the experiments and notes of salient changes were entered into the lab book but we did not obtain permanent records or carry out a quantitative analysis. As stated in the manuscript, the recording also showed muscle potentials, most importantly those associated with breathing. One change that we watch for is a transient increase in heart rate, accompanied by prolonged breathing muscle activity, immediately after a ketamine/xylazine injection. We believe this reflects slight hypoxia and avoided this

by adjusting the dosage if it occurred. Another salient change is a gradual overall increase of muscle potentials without any visible animal movement which signals that the plane of anaesthesia is getting lighter. We did not notice any other systematic changes in heart rate during a given experiment.

l.175 Here, or elsewhere, briefly describe the effect of non-venting the middle ear on thresholds

We have added a short explanation.

l.199 and following ... CAP and single unit recordings use a 5 Hz stimulus repetition rate, is

this also the case for ABR recordings?

It was very similar, at 7 Hz; this information is now added.

Did the scope of effects of anaesthesia depend on repetition rate? If auditory nerve or other peripheral adaptive phenomena differ for different anesthetics, repetition rate would influence results. It would also be valuable to assess whether evoked spike rates and latencies (response strength at some dB above threshold) differed between anesthesia groups.

Results may or may not be consistent with the authors' findings on ABR amplitude and

latency.

Considering the minor difference in stimulus repetition rates, we believe a differential effect is very unlikely. Brittan-Powell et al. (2002) tested the influence of stimulus repetition rate on ABR latency and amplitude in another bird, the budgerigar, under ketamine/diazepam anaesthesia. They found no change within the rates used in the present study; only at rates higher than 10 Hz, were significant increases in latency and decreases in amplitude observed.

l.239 It is not clear what advantages the ‘new’ measure of relative sensitivity - using Cap thresholds as reference for single unit thresholds - provides. Cap threshold data are obtained by much higher SPLs than the single unit thresholds Actual single unit thresholds in dB SPL would provide additional information on absolute auditory sensitivity in barn owls.

The original aim of the single-unit experiments on young owls was to study maturation of auditory-nerve responses. The age range of animals was chosen to include a period of significant maturation of auditory thresholds, as previously documented with CAP recordings (Köppl and Nickel, 2007). In contrast, the present manuscript focusses on anaesthesia effects on auditory sensitivity. The normalization of single-unit thresholds to the age-matched CAP audiogram was introduced to eliminate known maturation effects on thresholds and identify those changes that are due to the anaesthetic regime. We have added another explanatory sentence (line

277ff).

Absolute sensitivity has been published previously for barn owls, as psychophysical, auditory-nerve CAP and single-unit thresholds (Konishi, 1973; Dyson et al., 1998; Krumm et al., 2017; Köppl and Gleich, 2007; Köppl, 1997).

Statistical analyses: Spontaneous firing rates were significantly lower for the isoflurane- terminal group than the ketamine-terminal group overall, a finding of substantial relevance to this study. The statistical analysis reported in Fig. 4 seek to undercut this finding by breaking

the data down into bins by characteristic frequency. This grouping is justified by pointing out that spontaneous rates vary by CF, but sampling also varies between CF bins. The justification for an unconventional statistical approach is not clear. In Fig. 4, it is not clear how strong the main effect for anesthetic groups is; statistical comparisons between groups in smaller CF-specific samples could be corrupting this main effect. Notably individual Mann- Whitney U tests for comparisons between groups used as N as small 1 cell in the case of the high CF tuning group. It is likely that some of these tests lack the statistical power necessary to come to a reasonable CF-specific conclusion. A larger binning of CFs to improve sample sizes might help. Otherwise,main effect for the anesthetic group and the relatively large median decrease for the iso animals should be reported. A multivariate approach with consideration of multiple comparisons is also an alternative approach. A similar criticism is applicable to the statistical analysis performed on ABR thresholds reported in figure 6.

To emphasize the overall difference in spontaneous rates between the anaesthetic groups, we have added another panel to Fig. 4.

We do not seek to undercut any finding but simply present different ways of analysing the data which are both justified: grouped into CF-bins or pooled across all CFs. It is, of course, correct that a smaller sample size also means less statistical power, and that different binning of the data may lead to different results. However, bin sizes should be motivated by the known variation (here, the dependence of spontaneous rates on CF) and not by sample sizes. The statistical table lists all results and readers can judge for themselves which conclusion they find more convincing.

Discussion

Young owls in the ketamine-terminal group were induced and maintained under anesthesia with simple injections, while the isofurane-terminal animals underwent a tracheotomy and artificial respiration. The authors are correct to point out that it is not simply the drugs that differed in the two groups, but also the surgical procedures and maintenance of the plane of anesthesia that differed. Furthermore, most animals in the inhalant group were respirated with pure oxygen while the injection group breathed room air. To address the potential confounding factor of long-term exposure to pure oxygen, it would be valuable to see a comparison of the carbogen and oxygen groups.

We have added the requested comparison of ABR thresholds in adult owls, obtained while delivering inhalants in carbogen vs. delivery in pure oxygen (line 367 ff), and discuss it now more generally in the context of modes of respiration (not only artificial respiration) as a confounding factor (line 507 ff).

Our work identified important, previously unrecognized confounding effects of respiration, in addition to confirming a basic detrimental effect of inhalants. We feel unable to completely resolve all possible new aspects that were raised within the scope of this study but distil interesting questions for future research (see also next question).

The paper's impact would be strengthened by considering these results in the broader

context of neuronal processing at multiple levels of sensory hierarchies. For instance, what is the predicted impact of these levels of sensitivity loss on downstream regions, and what new insights does this potentially give us into the interpretation of previous data across avian or

even mammalian species? Finally, the authors are advised to address the bottom line here: what does the relative impact of gas anesthesia on neural responses say about its use in neurophysiology? Given that it has an observable dose-dependent effect, are there other ways of safely and humanely administering, for example, isoflurane while also mitigating depression of neural responses? One common approach is the combination of isoflurane and nitrous oxide, which allows for a deeper plane of anesthesia with less isoflurane and oxygen toxicity. Comparing this anesthetic regime with the others in this study would be highly valuable to the field.

We have added a section addressing these issues to the Discussion (line 546 ff).

Minor comments:

Line 42: “given neuronal population”

added

Line 262: The authors self-assign a significance criterion of p&lt;0.01, but report a significant difference of P&lt;0.03 in the text when describing Table 1, ref. #17.

Thank you for pointing this out. Statement has been corrected.

Line 319-20: The wording here is a little confusing. The ABR audiogram shapes were the same or the relationship between ABR threshold and Frequency had the same shape?

rephrased

## References

[B1] Anderson MJ, Young ED (2004) Isoflurane/N2O anesthesia suppresses narrowband but not wideband inhibition in dorsal cochlear nucleus. Hear Res 188:29–41. 10.1016/S0378-5955(03)00348-4 14759568

[B2] Antkowiak B (2001) How do general anaesthetics work? Naturwissenschaften 88:201–213. 11482433 10.1007/s001140100230

[B3] Bolhuis JJ, Okanoya K, Scharff C (2010) Twitter evolution: converging mechanisms in birdsong and human speech. Nat Rev Neurosci 11:747–759. 10.1038/nrn2931 20959859

[B4] Bremen P, Poganiatz I, Campenhausen M, Wagner H (2007) Sensitivity to interaural time difference and representation of azimuth in central nucleus of inferior colliculus in the barn owl. J Comp Physiol A 193:99–112. 10.1007/s00359-006-0172-z 17021830

[B5] Burger RE, Lorenz FW (1960) Artificial respiration in birds by unidirectional air flow. Poultry Sci 39:236–237. 10.3382/ps.0390236

[B6] Burns P (2014) Isoflurane & sevoflurane: mechanics & effects. Clin Brief 23–26. Available at https://www.cliniciansbrief.com/article/isoflurane-sevoflurane-mechanics-effects

[B7] Campagna JA, Miller KW, Forman SA (2003) Mechanisms of actions of inhaled anesthetics. N Engl J Med 348:2110–2124. 10.1056/NEJMra021261 12761368

[B8] Cederholm JM, Froud KE, Wong AC, Ko M, Ryan AF, Housley GD (2012) Differential actions of isoflurane and ketamine-based anaesthetics on cochlear function in the mouse. Hear Res 292:71–79. 10.1016/j.heares.2012.08.010 22960466 PMC3582347

[B9] Cheung SW, Nagarajan SS, Bedenbaugh PH, Schreiner CE, Wang X, Wong A (2001) Auditory cortical neuron response differences under isoflurane versus pentobarbital anesthesia. Hear Res 156:115–127. 10.1016/S0378-5955(01)00272-6 11377887

[B10] Cohen YE, Knudsen EI (1995) Binaural tuning of auditory units in the forebrain archistriatal gaze fields of the barn owl: local organization but no space map. J Neurosci 15:5152–5168. 7623142 10.1523/JNEUROSCI.15-07-05152.1995 PMC6577861

[B11] Desmarchelier M, Rondenay Y, Fitzgerald G, Lair S (2007) Monitoring of the ventilatory status of anesthetized birds of prey by using end-tidal carbon dioxide measured with a microstream capnometer. J Zoo Wildl Med 38:1–6. 10.1638/05-033.1 17469268

[B12] Dodd F, Capranica RR (1992) A comparison of anesthetic agents and their effects on the response properties of the peripheral auditory system. Hear Res 62:173–180. 10.1016/0378-5955(92)90183-N 1429259

[B13] Drexl M, Henke J, Kössl M (2004) Isoflurane increases amplitude and incidence of evoked and spontaneous otoacoustic emissions. Hear Res 194:135–142. 10.1016/j.heares.2004.04.006 15276684

[B14] Eatock RA, Lysakowski A (2006) Mammalian vestibular hair cells In: Vertebrate hair cells (Eatock RA, Fay RR, Popper AN, eds), pp 348–442. New York: Springer.

[B15] Ferber-Viart C, Preckel MP, Dubreuil C, Banssillon V, Duclaux R (1998) Effect of anesthesia on transient evoked otoacoustic emissions in humans: a comparison between propofol and isoflurane. Hear Res 121:53–61. 9682807 10.1016/s0378-5955(98)00064-1

[B16] Flaherty D (2009) Anaesthetic drugs In: Anaesthesia for veterinary nurses (Welsh L, ed), pp 121-161 Chichester, UK: Wiley-Blackwell.

[B17] Glowatzki E, Grant L, Fuchs P (2008) Hair cell afferent synapses. Curr Opin Neurobiol 18:389–395. 10.1016/j.conb.2008.09.006 18824101 PMC2860955

[B18] Gungor G, Bozkurt-Sutas P, Gedik O, Atas A, Babazade R, Yilmaz M (2015) Effects of sevoflurane and desflurane on otoacoustic emissions in humans. Eur Arch Otorhinolaryngol 272:2193–2199. 10.1007/s00405-014-3124-1 25027945

[B19] Gunkel C, Lafortune M (2005) Current techniques in avian anesthesia. Semin Avian Exotic Pet Med 14:263–276. 10.1053/j.saep.2005.09.006

[B20] Ishizawa Y (2007) Mechanisms of anesthetic actions and the brain. J Anesth 21:187–199. 10.1007/s00540-006-0482-x 17458649

[B21] Jaensch S, Cullen L, Raidal SR (2001a) Normobaric hyperoxic stress in budgerigars: enzymic antioxidants and lipid peroxidation. Comp Biochem Physiol C Toxicol Pharmacol 128:173–180. 10.1016/S1532-0456(00)00186-1 11239830

[B22] Jaensch S, Cullen L, Morton L, Raidal SR (2001b) Normobaric hyperoxic stress in budgerigars: non-enzymic antioxidants. Comp Biochem Physiol C Toxicol Pharmacol 128:181–187. 10.1016/S1532-0456(00)00187-3 11239831

[B23] Katz E, Elgoyhen AB, Fuchs PA (2011) Cholinergic inhibition of hair cells In: Auditory and vestibular efferents (Ryugo D, Fay RR, Popper AN, eds), pp 103–133. New York: Springer.

[B24] Keegan RD (2005) Inhalant anesthetics: the basics In: Recent advances in veterinary anesthesia and analgesia: companion animals (Gleed RD, Ludders JW, eds). Ithaca, NY: International Veterinary Information Service.

[B25] Keller CH, Takahashi TT (2005) Localization and identification of concurrent sounds in the owl's auditory space map. J Neurosci 25:10446–10461. 10.1523/JNEUROSCI.2093-05.2005 16280583 PMC6725814

[B26] Kettembeil S, Manley GA, Siegl E (1995) Distortion-product otoacoustic emissions and their anaesthesia sensitivity in the European Starling and the chicken. Hear Res 86:47–62. 8567421 10.1016/0378-5955(95)00053-7

[B27] Köppl C (1997) Frequency tuning and spontaneous activity in the auditory nerve and cochlear nucleus magnocellularis of the barn owl, *Tyto alba*. J Neurophysiol 77:364–377. 10.1152/jn.1997.77.1.364 9120577

[B28] Köppl C (2011) Evolution of the octavolateral efferent system In: Auditory and vestibular efferents (Ryugo D, Fay RR, Popper AN, eds), pp 217–259. New York: Springer.

[B29] Köppl C, Gleich O (2007) Evoked cochlear potentials in the barn owl. J Comp Physiol A Neuroethol Sens Neural Behav Physiol 193:601–612. 10.1007/s00359-007-0215-0 17318655

[B30] Köppl C, Nickel R (2007) Prolonged maturation of cochlear function in the barn owl after hatching. J Comp Physiol A Neuroethol Sens Neural Behav Physiol 193:613–624. 10.1007/s00359-007-0216-z 17323066

[B31] Köppl C, Futterer E, Nieder B, Sistermann R, Wagner H (2005) Embryonic and posthatching development of the barn owl (*Tyto alba*): reference data for age determination. Dev Dyn 233:1248–1260. 10.1002/dvdy.20394 15861405

[B32] Korbel R (1998) Vergleichende Untersuchungen zur Inhalationsanästhesie mit Isofluran (Forene) und Sevofluran (SEVOrane) bei Haustauben (Columba livia Gmel., 1789, var. domestica) und Vorstellung eines Referenz-Narkoseprotokolls für Vögel. Tierärztliche Praxis 26:211–223. 9646418

[B33] Larsen ON, Christensen-Dalsgaard J, Jensen KK (2016) Role of intracranial cavities in avian directional hearing. Biol Cybern 110:319–331. 10.1007/s00422-016-0688-4 27209199

[B34] Lierz M, Korbel R (2012) Anesthesia and analgesia in birds. J Exotic Pet Med 21:44–58. 10.1053/j.jepm.2011.11.008

[B35] Lukasik VM, Gillies RJ (2003) Animal anaesthesia for in vivo magnetic resonance. NMR Biomed 16:459–467. 10.1002/nbm.836 14696002

[B36] Michelson NJ, Kozai TDY (2018) Isoflurane and ketamine differentially influence spontaneous and evoked laminar electrophysiology in mouse V1. J Neurophyiol. Advance online publication. Retrieved October 27, 2018. doi:10.1152/jn.00299.2018. 10.1152/jn.00299.2018 PMC6295540 30067128

[B37] Noda T, Takahashi H (2015) Anesthetic effects of isoflurane on the tonotopic map and neuronal population activity in the rat auditory cortex. Eur J Neurosci 42:2298–2311. 10.1111/ejn.13007 26118739

[B38] O'Keeffe NJ, Healy TEJ (1999) The role of new anesthetic agents. Pharmacol Ther 84:233–248. 10665829 10.1016/s0163-7258(99)00034-0

[B39] Powell FL (2015) Respiration In: Sturkie’s avian physiology, Vol6 (Scanes CG, ed), pp 301–336. Amsterdam: Elsevier.

[B40] Preckel B, Bolten J (2005) Pharmacology of modern volatile anaesthetics. Best Pract Res Clin Anaesthesiol 19:331–348. 16013685 10.1016/j.bpa.2005.01.003

[B41] Raftery A (2013) Avian anaesthesia. In Pract 35:272–278. 10.1136/inp.f2861

[B42] Ropposch T, Walch C, Avian A, Mausser G, Spary M (2014) Effects of the depth of anesthesia on distortion product otoacoustic emissions. Eur Arch Otorhinolaryngol 271:2897–2904. 10.1007/s00405-013-2780-x 24150547

[B43] Rudolph U, Antkowiak B (2004) Molecular and neuronal substrates for general anaesthetics. Nat Rev Neurosci 5:709–720. 10.1038/nrn1496 15322529

[B44] Ruebhausen MR, Brozoski TJ, Bauer CA (2012) A comparison of the effects of isoflurane and ketamine anesthesia on auditory brainstem response (ABR) thresholds in rats. Hear Res 287:25–29. 10.1016/j.heares.2012.04.005 22543090

[B45] Ruel J, Wang J, Rebillard G, Eybalin M, Lloyd R, Pujol R, Puel JL (2007) Physiology, pharmacology and plasticity at the inner hair cell synaptic complex. Hear Res 227:19–27. 10.1016/j.heares.2006.08.017 17079104

[B46] Schwartzkopff J, Brémond JC (1963) Méthode de dérivation des potentiels cochléaires chez l`oiseau. J Physiol Paris 55:495–518. 14082131

[B47] Shawyer CR (1998) The barn owl. Chelmsford, UK: Arlequin.

[B55] Sloan T, Sloan H, Rogers J (2010) Nitrous oxide and isoflurane are synergistic with respect to amplitude and latency effects on sensory evoked potentials. J Clin Monit Comput 24:113–123. 10.1007/s10877-009-9219-3 20063047

[B48] Smith DI, Mills JH (1989) Anesthesia effects: auditory brain-stem response. Electroencephal Clin Neurophysiol 72:422–428. 2469566 10.1016/0013-4694(89)90047-3

[B49] Smith PH, Spirou GA (2002) From the cochlea to the cortex and back In: Integrative functions in the mammalian auditory pathway (Oertel D, Fay RR, Popper AN, eds), pp 6–71. New York: Springer Verlag.

[B50] Sonner JM, Antognini JF, Dutton RC, Flood P, Gray AT, Harris RA, Homanics GE, Kendig J, Orser B, Raines DE, Trudell J, Vissel B, Eger EI (2003) Inhaled anesthetics and immobility: mechanisms, mysteries, and minimum alveolar anesthetic concentration. Anesth Analg 97:718–740. 12933393 10.1213/01.ANE.0000081063.76651.33

[B51] Stronks HC, Aarts MCJ, Klis SFL (2010) Effects of isoflurane on auditory evoked potentials in the cochlea and brainstem of guinea pigs. Hear Res 260:20–29. 10.1016/j.heares.2009.10.015 19878711

[B52] Vahle-Hinz C, Detsch O (2002) What can in vivo electrophysiology in animal models tell us about mechanisms of anaesthesia? Br J Anaesth 89:123–142. 12173225 10.1093/bja/aef166

[B53] Varner J, Clifton KR, Broderson R, Wyatt RD (2004) Lack of efficacy of injectable ketamine with xylazine or diazepam for anesthesia in chickens. Lab Anim 33:36–39. 10.1038/laban0504-36 15141245

[B54] Windels F (2006) Neuronal activity: from in vitro preparation to behaving animals. Mol Neurobiol 34:1–25. 17003519 10.1385/mn:34:1:1

